# Timing of intubation in COVID-19: Not just location, location, location?

**DOI:** 10.1186/s13054-021-03617-2

**Published:** 2021-06-04

**Authors:** David A. Thomson, Gregory L. Calligaro

**Affiliations:** 1 Division of Critical Care, Department of Anaesthesia and Perioperative Medicine, University of Cape Town and Groote Schuur Hospital, Cape Town, South Africa; 2 Centre for Lung Infection and Immunity, Division of Pulmonology, Department of Medicine and UCT Lung Institute and South African MRC/UCT Centre for the Study of Antimicrobial Resistance, University of Cape Town, Cape Town, South Africa

We read with interest the paper by Papoutsi *et. al.* in which they synthesise the available evidence for early versus late intubation for COVID-19-related respiratory failure [1]. While many studies have reported on predictors of mortality in severe COVID-19, the majority of these factors are not modifiable. The selection for and timing of the escalation to mechanical ventilation are modifiable and of particular relevance in resource-constrained settings to optimise the use of ICU resources [2].

Differentiating between the timing of intubation once admitted to ICU for 24 h (the primary definition in their study) or intubation based on a trial of non-invasive mechanical support (their secondary definition) has vastly different implications. At our institution, a decision was made to reserve and expand ICU services only for COVID-19 patients requiring invasive mechanical ventilation, and to offer high-flow nasal oxygen (HFNO) in a non-critical care ward-based environment. Patients coming to the ICU were all intubated outside of the ICU having had a trial of HFNO [3]. Our patients would be considered early intubations by the primary definition, and late intubations in the secondary analysis.

In our cohort of 293 patients, we had 52.7% survival without mechanical ventilation (with a median P/F ratio of 68 on non-rebreather support at initiation of HFNO) and a median duration of HFNO support of 6 days in survivors, and 2 days in non-survivors. Unfortunately, this result needs to be balanced against the initial poor outcomes (8% survival) of those patients who progressed to need invasive mechanical ventilation [4]. We would therefore caution against a definition of early intubation that is based on the patient accessing a physical location (ICU) or level of support (HFNO) without quantifying the severity of the patient’s underlying respiratory failure or need for other organ support.

It is essential that studies provide consistent information on the severity of illness and information is given on context and setting when reporting studies on mechanical ventilation in COVID-19 pneumonia. We noted that 5 of the 12 studies did not report the SOFA score of the patients at the time of ICU admission and that 6 of the 12 publications analysed required the authors to supply additional information from that published in order to allow this analysis. Understanding the clinical triggers for accessing ICU care, the severity of respiratory failure prior to intubation, and the available resources in a particular setting is key to assessing time-sensitive interventions such as intubation.

## Data Availability

Patient data has been previously published and is available on request [4].

## References

[1] Papoutsi E, Giannakoulis VG, Xourgia E, Routsi C, Kotanidou A, Siempos II (2021). Effect of timing of intubation on clinical outcomes of critically ill patients with COVID-19: a systematic review and meta-analysis of non-randomized cohort studies. Crit Care.

[2] Morrow BM, Gopalan PD, Joubert I, Paruk F, Pope A (2020). Critical care triage during the COVID-19 pandemic in South Africa: A constitutional imperative!. SAMJ S Afr Med J.

[3] Mendelson M, Boloko L, Boutall A, Cairncross L, Calligaro G, Coccia C, Dave JA, De Villiers M, Dlamini S, Frankenfeld P, Gina P (2020). Clinical management of COVID-19: experiences of the COVID-19 epidemic from Groote Schuur Hospital, Cape Town, South Africa. SAMJ S Afr Med J.

[4] Calligaro GL, Lalla U, Audley G, Gina P, Miller MG, Mendelson M, Dlamini S, Wasserman S, Meintjes G, Peter J, Levin D (2020). The utility of high-flow nasal oxygen for severe COVID-19 pneumonia in a resource-constrained setting: a multi-centre prospective observational study. EClinicalMedicine.

